# Posterior compartment symptoms in primiparous women 1 year after non-assisted vaginal deliveries: a Swedish cohort study

**DOI:** 10.1007/s00192-021-04700-6

**Published:** 2021-03-01

**Authors:** Emilia Rotstein, Susanne Åhlund, Helena Lindgren, Angelica Lindén Hirschberg, Ingela Rådestad, Gunilla Tegerstedt

**Affiliations:** 1 Karolinska Pelvic Floor Centre, Karolinska University Hospital Huddinge, Stockholm, Sweden; 2 Department of Clinical Science, Intervention and Technology (CLINTEC), Karolinska Institutet, 171 77 Solna, Sweden; 3 Department of Women’s and Children’s Health, Karolinska Institutet, Stockholm, Sweden; 4 Department of Gynaecology and Reproductive Medicine, Karolinska University Hospital, Stockholm, Sweden; 5 Sophiahemmet University, Stockholm, Sweden

**Keywords:** Incontinence, Perineum, Urogynaecology

## Abstract

**Introduction and hypothesis:**

This is a prospective cohort follow-up study based on the hypothesis that primiparous women with non-assisted vaginal deliveries and a second-degree perineal tear have more posterior compartment symptoms 1 year after delivery than those with no or first-degree tears.

**Methods:**

A follow-up questionnaire, including validated questions on pelvic floor dysfunction, was completed 1 year postpartum by 410 healthy primiparas, delivered without instrumental assistance at two maternity wards in Stockholm between 2013 and 2015. Main outcome measures were posterior compartment symptoms in women with second-degree perineal tears compared with women with no or only minor tears.

**Results:**

Of 410 women, 20.9% had no or only minor tears, 75.4% had a second-degree tear, and 3.7% had a more severe tear. Of women presenting with second-degree tears, 18.9% had bowel-emptying difficulties compared with 20.0% of women with minor tears. Furthermore, almost 3% of them with second-degree tears complained of faecal incontinence (FI) of formed stool, 7.2% of FI of loose stool compared with 1.2% and 3.5% respectively in women with no or only minor tears.

**Conclusions:**

Symptomatic pelvic floor dysfunction is common among primiparous women within 1 year following uncomplicated vaginal delivery, and there are no significant differences between second-degree perineal tears and minor tears. These symptoms should be addressed in all women after delivery to improve pelvic floor dysfunction and quality of life.

## Introduction

Vaginal delivery may lead to various short- and long-term pelvic floor disorders, such as urinary incontinence (UI), faecal incontinence (FI) and pelvic organ prolapse [1], especially after instrumentally assisted deliveries or deliveries resulting in obstetric anal sphincter injuries (OASIs) [2]. This is an inevitable component of vaginal delivery; however, the scientific focus has been on the minority acquiring major perineal trauma and not on the majority, who deliver unassisted with no more than a second-degree perineal tear [3].

The majority of primiparae births will result in a second-degree tear [4], injuring the perineal body that forms an important link between the levator ani muscle hammock and the rectovaginal fascia, as well as the transverse perineal muscles, the anal sphincter complex and the bulbocavernosus muscle.

Definitions of perineal tears are primarily focused on OASI, even though second-degree tears may also range from quite miniscule to highly complicated tears. A second-degree tear is considered atraumatic following delivery, although it has been shown to be a risk factor for occult OASI [5]. Previously described risk factors for perineal tears include maternal age, foetal birthweight, and head circumference [6–9]. Primiparous women are at the highest risk of at least a second-degree tear [10], yet the symptoms these women display 1 year postpartum are not well described [3].

The objective of this prospective cohort follow-up study was to evaluate posterior compartment symptoms 1 year after delivery in primiparous women with non-assisted vaginal deliveries and to compare symptoms in women with second-degree perineal tears with those in women with no or minor tears. The hypothesis is that a second-degree perineal tear might harm the functional unit of the perineal body to a greater extent than previously described. As such, subtypes of FI were analysed as secondary outcomes in this study.

## Materials and methods

### Cohort

**Fig. 1:**
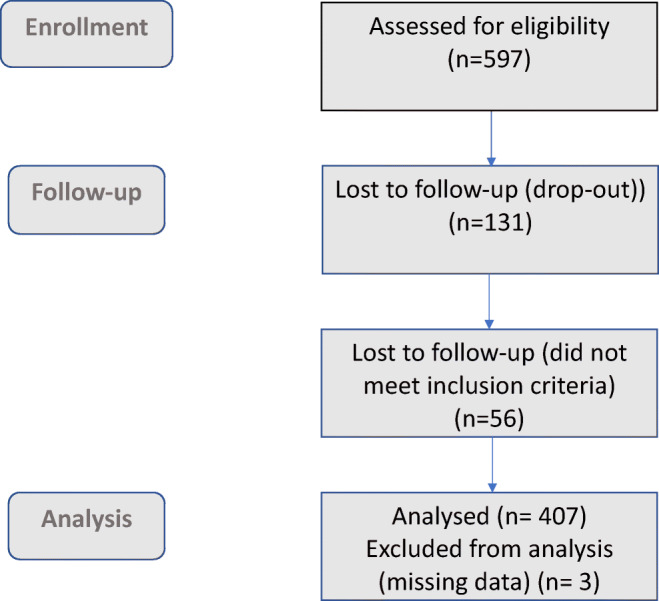
Flow diagram

This cohort study is a follow-up from the MIMA (Midwives’ Management during the second stage of labour) study [11], an interventional cohort study, conducted in two delivery wards in Stockholm between 2013 and 2015. The underlying MIMA study included 597 primiparous Swedish-speaking women, with spontaneous onset of labour or induction of labour at the gestational age of ≥ 37 full weeks of pregnancy. Of these, 466 completed the 1-year follow-up questionnaire, and 56 of those did not meet the inclusion criteria owing to novel pregnancies. Thus, our study population comprised 410 women with a drop-out rate of three women because of missing data on perineal tear. A flowchart of the population is shown in Fig. 1. Women with diabetes mellitus (gestational or manifest), female genital mutilation, intrauterine growth restriction, stillbirth, breech presentation, instrumental birth, multiple pregnancy or a new pregnancy within 1 year postpartum were excluded. Women who met the inclusion criteria were asked to participate on admission to the delivery ward. There was no access to information of potential previous symptoms of pelvic floor dysfunction before or during pregnancy in these women.

### Ethical approval

Ethics approval for the study was granted by the Regional Ethics Review Board at Karolinska Institutet (2013/859–31/2). All women included in the study signed written consent according to the ethical approval of the study.

### Obstetric variables

Demographic data such as age, level of education, body mass index (BMI), marital status, and tobacco use were attained from the questionnaire, whereas delivery information was retrieved from obstetric charts in the local hospital database after signed consent as part of the questionnaire. Continuous variables were categorised including BMI (< 18.5, 18.5—24.9, 25.0—29.9, ≥ 30), age (< 25, 25—35, > 35) and foetal birth weight (< 3000 g, 3000–3499 g, 3500–4000 g, > 4000 g) and head circumference (< 34.9 cm, ≥ 35 cm). Upright positions were defined as positions maximising the pelvic outlet measurements (standing, kneeling, on all fours, birthing seat, or lateral lying position) whereas semi-recumbent and lithotomy positions were considered as supine positions [11].

Perineal injuries were categorised according to international standards into grade 0–1, grade 2 and grade 3 + 4. Each perineal tear was assessed directly following birth by two examiners describing it in words, measuring it in three dimensions (height, length and depth) and by marking it on a schematic image using a set protocol. These descriptions and protocols were re-evaluated by an independent examiner (midwife) at a later point in time and, in cases of uncertainty, the measurements and schematic images were discussed with two urogynaecologists assessing them together with the independent examiner. The category 0–1 included no tear, labial tear only, tears of the perineal skin and/or tears involving the vaginal mucosa no deeper than 0.5 cm. If the vaginal tear exceeded 0.5 cm, it was classified as a second-degree tear as it was likely to involve the rectovaginal fascia, as were all episiotomies.

In this study, all second-degree tears were analysed and compared with those with minor tears, i.e. no to first-degree tears. We hypothesised that the tears labelled as no tear or first degree did not involve the rectovaginal fascia or muscular structures of the perineum, thus keeping the functional unit of the distal pelvic floor intact, and they were considered as reference. All second-degree tears, however, were assumed to involve the rectovaginal fascia or muscular structures of the perineum and were defined as the exposure group. The category grade 3 + 4 included all tears affecting the anal sphincter complex, were not included in statistical analyses and are only reported for descriptive comparison.

### Study questionnaire

A questionnaire with validated questions [12, 13] covering known and suggested persistent symptoms of pelvic floor dysfunction was sent by postal carrier to all participating women 12 months after delivery. The questionnaire included different areas of pelvic floor dysfunction, such as FI and bowel-emptying difficulties, using parts of the Hvidovre hospital validated questionnaire, as well as the PFIQ-7 and PFDI-20 [13, 14]. The questions were aimed at targeting symptoms experienced in the preceding 3 months. Face-to-face validation of the questionnaire had been performed with 12 women, after which minor changes were made. The women received one reminder to respond and if they did not, they were excluded.

A response of yes to the question “incontinence of formed stool” and/or “incontinence of loose stool” in the study questionnaire was regarded as having symptoms of FI. The inability to restrain the leakage of gas anally was regarded as flatus incontinence. A response of yes to questions regarding difficulties emptying the bowel in general or the need to digitally assist bowel emptying specifically were both considered as bowel-emptying difficulties. The issues of painful defecation and a sense of bulging in or outside of the vaginal opening were also addressed. The terminology in this manuscript follows the International Urogynecological Association (IUGA)/International Continence Society (ICS) Joint Report on Terminology for Female Pelvic Floor Dysfunction where applicable [15].

“Sexual complaints” were defined as answering at least “a little bit” to any of the questions addressing this issue, i.e. including sensory complains, pain and altered orgasm.

### Statistical analyses

Descriptive analysis (n, percentage, mean) and Pearson Chi-squared test were used to present background and compare obstetric characteristics and posterior compartment symptoms between women with second-degree perineal tears and those with no or minor tears. p values lower than 0.05 were considered statistically significant. The IBM SPSS Statistics for MacIntosh/Windows (version 24.0; SPSS, Chicago, IL, USA) was employed for the data analysis.

## Results

For this study, 410 women were found to be eligible and responded to the questionnaire (68.7%), 3 of those were considered to be drop-outs owing to missing data on perineal tears. Mean age at delivery was 29.6 years (total range 17–45 years, SD 4.5), and the mean BMI was 23.0 (16.9–39.4, SD 3.4). Over two-thirds (71.2%) reported a college- or university-level education, and almost none used tobacco (3.4%, Table 1). There were no statistically significant differences between the groups regarding socio-demographic characteristics.

**Table 1:** Socio-demographic background among 407 women in Sweden, 1 year postpartum^a^

	Minor injury (none to first degree) *n* = 85	Second degree *n* = 307	Severe injury (third to fourth degree) *n* = 15
Age	28.7 ± 5.2	30.0 ± 4.3	28.8 ± 3.6
Marital status			
Single	4 (4.3)	7 (2.3)	0 (0.0)
Married/co-habiting	80 (95.2)	298 (97.7)	14 (100.0)
Smoking at first antenatal visit	2 (2.4)	3 (1.0)	0 (0.0)
BMI 1 year-postpartum	23.4 ± 6.2	22.9 ± 3.6	24.1 ± 6.3
<18.5	4 (5.0)	10 (3.3)	0 (0.0)
18.5–24.9	60 (75.0)	210 (68.4)	11 (84.6)
25.0–29.9	11 (13.8)	55 (17.9)	0 (0.0)
>30	5 (6.2)	7 (2.3)	2 (15.4)
Level of education			
Elementary school or upper secondary	33 (38.8)	71 (23.5)	7 (53.8)
University or college degree	52 (61.2)	231 (76.5)	6 (46.2)
Health-related problems during pregnancy	7 (8.2)	40 (13.0)	0 (0.0)

Values presented as numbers (%) or mean ± SD

^a^ Total number less than 407 indicates missing data

The obstetric and birth characteristics are presented in Table 2. There was no statistically significant difference between the women presenting with no to minor perineal tears when compared with those diagnosed with second-degree tears. Episiotomies (2.6%) were classified as second-degree tears.

**Table 2:** Obstetric and birth characteristics among 407 women in Sweden, 1-year postpartum^a^

	Minor injury (none to first degree) *n* = 85	Second degree *n* = 307	Severe injury (third to fourth degree) *n* = 15
Induction of labour			
Spontaneous	70 (82.4)	262 (85.6)	13 (86.7)
Induction	15 (17.6)	44 (14.4)	2 (13.3)
Epidural analgesia	53 (62.4)	171 (55.7)	8 (53.3)
Oxytocin during active second stage	6 (7.1)	33 (10.7)	2 (13.3)
Passive second stage (min)			
<180 min	80 (95.2)	262 (90.0)*	9 (69.2)
>180 min	4 (4.8)	29 (10.0)	4 (30.8)
Active second stage (min)			
<60 min	83 (98.8)	280 (96.2)	12 (92.3)
>60 min	1 (1.2)	11 (3.8)	1 (7.7)
Birth position			
Upright	31 (36.5)	118 (38.4)	6 (40.0)
Supine	54 (63.5)	189 (61.6)	9 (60.0)
Presentation			
OAP	82 (96.5)	297 (96.7)	14 (93.3)
Non-OAP	3 (3.5)	10 (3.3)	1 (6.7)
Birth weight (g)	3,458.5 ± 376.2	3,499.0 ± 400.0	3,684.8 ± 509.2
Head circumference (cm)	34.6 ± 1.3	34.8 ± 1.4	34.8 ± 1.4
>35 cm	39 (45.9)	160 (52.1)	7 (46.7)

Values presented as numbers (%) or mean (±SD)

*p value <0.05 based on Chi-squared test for dichotomous variables, t test for normally distributed continuous variables and the Mann–Whitney U test for ordinal or non-normally distributed variables; comparing minor injury with second-degree injury

^a^ Total number less than 407 indicates missing data

The prevalence of posterior compartment symptoms for the women with second-degree tears was 18.9% for bowel-emptying difficulties, 7.2% and 2.9% for incontinence of loose and formed stool respectively, and 38.4% reported flatus incontinence (Table 3). Faecal urgency and FI during sexual intercourse for these women with second-degree tears were 19.9% and 1.6% respectively, and the corresponding figures for women with no to first-degree tears were 20.0% (bowel-emptying difficulties, 1.2% (FI formed stool), 3.5% (FI loose stool), 32.9% (FI flatus), 21.2% (faecal urgency) and none had FI during sexual intercourse. There were no statistically significant differences between the respondents with minor tears and those with second-degree tears concerning any outcome measures (Table 3).

**Table 3:** Posterior compartment symptoms among 407 women in Sweden, 1 year postpartum

	Minor injury (none to first degree) *n* = 85	Second degree *n* = 307	Severe injury (third to fourth degree) *n* = 15
FI formed stool	1 (1.2)	9 (2.9)	0 (0.0)
FI loose stool	3 (3.5)	22 (7.2)	1 (6.7)
FI flatus	28 (32.9)	118 (38.4)	7 (46.7)
FI with vaginal intercourse	0 (0.0)	5 (1.6)	1 (6.7)
Faecal urgency	18 (21.2)	61 (19.9)	4 (26.7)
Pain with defecation	14 (16.5)	67 (21.8)	3 (20.0)
Bowel-emptying difficulty	17 (20.0)	58 (18.9)	4 (26.7)
Need for manual digitation	10 (11.8)	26 (8.5)	0 (0.0)
Bulging sensation	6 (7.1)	25 (8.1)	1 (6.7)
Sexual complaints	38 (44.7)	163 (53.1)	12 (80.0)

Values presented as numbers (%) or mean (±SD)

*p value < 0.05 based on Chi-squared test for dichotomous variables, t test for normally distributed continuous variables and the Mann–Whitney U test for ordinal or non-normally distributed variables; comparing minor injury with second-degree injury

There were no statistically significant differences between women who completed the questionnaire and those who did not regarding BMI, the severity of perineal injury, duration of the second stage, birth position or the baby’s birth weight and head circumference. However, we found that there was a statistically significant difference between responders and non-responders with regard to age and smoking habits; the non-responders were younger and the smokers smoked to a greater extent.

## Discussion

In this study, with a prospective cohort design including primiparous women in an uncomplicated vaginal birth setting, we aimed to compare posterior compartment symptoms in women with no to minor perineal tears with those with second-degree tears. Although our hypothesis was that second-degree tears may cause symptoms that are more severe than minor tears was not proven, we found that roughly 1 in 5 of responders within both groups compared displayed symptoms of bowel-emptying difficulties and about 1% of women with no to minor tears and 3% of women with second-degree tears experienced incontinence of formed stool 1 year after delivery.

The rectovaginal fascia is often involved in a perineal injury. It attaches to the perineal body and is vital in the anterior support of the anal canal [16, 17], and although we hypothesised that an injury of the muscular and deeper connective tissue layers of the pelvic floor plays an important role in the functionality of defecation, this study has not ascertained this. Rather, it could very well be the pregnancy itself that contributes to these complaints, or another factor not studied, such as a levator ani muscle injury [18–20], or overdistension of the supportive connective tissues or nerves of the pelvic floor complex. Even though we found no statistically significant difference in the reference and exposed groups concerning FI, there is still a striking number of women with some degree of FI in both groups generally considered as minor perineal trauma, whereas previous studies have focused mainly on major perineal trauma [7, 21, 22].

It is known that a majority of women obtain some degree of perineal tear during vaginal delivery [10, 23], and primiparous women experience the highest risk of suffering more severe perineal tears [24, 25]. Second-degree tears can in the worst cases cause problems similar to those due to anal sphincter tears. Our findings demonstrate that women with minor and second-degree tears have complaints of pelvic floor disorders comparable with or more severe than those with obstetric anal sphincter tears described in previous studies, and we believe that there is a need to address issues other than FI and obstetric anal sphincter injuries when counselling women postpartum. Our main findings emphasise the fact that not only obstetric anal sphincter tears have severe symptoms of posterior compartment deficiency. There are women in all groups of perineal tears in our study who responded as having FI, which to our knowledge has not been previously reported.

In Sweden, the option of a postnatal consultation is voluntary and falls on the woman to make the appointment, and in 2014, over three-quarters (77%) of women made the appointment. Furthermore, the appointment is with a midwife, and rarely with the caregiver who managed the delivery. In an Italian study by Soligo et al. [25], only approximately half of women were invited back for consultation and assessment of pelvic floor dysfunction and at a tertiary referral hospital in Milan 3 months postpartum, and this was in a study setting with multiple reminders. Furthermore, it has been shown that only 33% of international urogynaecologists and 25% of obstetricians routinely counsel women on the prevention of postnatal pelvic floor dysfunction, even though 60% of them are aware of the major risk factors and protective factors [26]. Lipschuetz et al. [27] state that 64% of primiparous women report at least one symptom of a pelvic floor disorder, making it a major concern postpartum. Faecal incontinence is a significant factor that decreases quality of life [28, 29], and must thus be properly assessed and addressed postnatally.

Consistent with de Leeuw et al. [30], the vast majority of women who undergo vaginal delivery will not obtain an OASI. Other studies in similar populations have reported a higher incidence of FI in women postpartum. Handa et al. [31] reported an incidence of 30.6% in a 15-year postpartum follow-up. Similarly, a register-based study published in The Lancet in 2019 [32] reported an incidence of FI of 37% postpartum, additionally stating high maternal age and foetal bodyweight as the strongest risk factors. Rikard-Bell et al. [33] failed to demonstrate any statistically significant differences between perineal outcomes and symptoms of bowel dysfunction, prolapse or sexual dysfunction. Furthermore, in 2016, Leeman et al. [34] published a prospective study where rates of FI did not differ between groups (7% for intact/minor laceration vs 10% for second-degree or greater laceration). They conclude that "women having second degree laceration are not an increased risk for pelvic floor dysfunction other than increased pain, and slightly lower sexual function scores at 6 months postpartum." Here, we have studied specific features of FI in a selected group of women with minor and second-degree injuries only. We would argue that in this way our findings add even more evidence that women with no perineal tears also suffer from pelvic floor dysfunction of the magnitude that was previously considered to be linked primarily to third- and fourth-degree perineal tears.

We hypothesised from these data that second-degree tears should be paid far more attention as far as diagnostics and suturing technique is concerned, and addressing possible complaints and symptoms of pelvic floor dysfunction postpartum. Anatomically, one might argue that the anterior and lateral support to the anal canal is mainly formed by the rectovaginal fascia and the pubococcygeal muscle, emphasising the importance of these structures in preventing the symptoms previously described. This support and its innervation are crucial for the functionality of defecation, and as we have identified a number of women with minor tears who reported having FI, we suggest that these structures might be affected even when no actual tear is present, possibly suggesting that distension and concomitant affection of innervation may be involved. However, the focus of caregivers has been on tears involving the anal sphincter complex, thus leaving many women who seek counsel to suffer unnecessarily without being understood or helped.

An issue that may interfere with the cohort is the general way in which maternal care and deliveries are managed in Sweden, as midwives are the primary caregivers at the labour ward, although there is close interaction with the attending physicians. Midwives manage uncomplicated deliveries of low-risk patients including the diagnostics and suturing of first- and second-degree perineal tears, whereas generally, complicated deliveries including instrumentally assisted births and severe perineal tears are the responsibility of the on-call obstetricians and gynaecologists. We have tried to minimise this bias by the examination methodology described in the Materials and methods section above. In recent years, there has been a large national enterprise for disseminating the knowledge and skills of diagnostics and perineal repair postpartum.

Our findings lead to the conclusion that minor or second-degree tears may very well mimic and sometime surpass OASI as far as posterior compartment symptoms are concerned. An unexpectedly high number of the women in our study presented with symptoms previously seen as exclusively related to major obstetric trauma and instrumentally assisted deliveries. Although the response frequency was high, the study was limited by the fact that further data on suturing techniques and postpartum wound healing were not obtained or analysed. It could also be argued that other limitations of our study are the fact that 71.2% of the women reported a college- or university-level education. This kind of selection bias can be attributed to the recruitment and the location in Stockholm of the original study groups, which also failed to register ethnicity and a possible factor affecting the replies to the questions posed. Having such a highly educated group of participants may play a role in the responses received, along with other demographic features, such as a mean BMI of 23, which is not representative of birthing women in Stockholm, and would suggest that our results may not be directly or fully applicable in other populations.

## Conclusion

A high proportion of women have bowel-emptying difficulties and FI, including soiling and flatus incontinence after an uncomplicated delivery of their first child. No statistically significant difference with regard to no or second-degree perineal injury was found in this study. However, the rate of symptoms 1 year after delivery was comparable with those who sustained anal sphincter injuries [21, 35, 36]. We propose focussing on diligent education and perineal evaluation postpartum, as well as information to patients, in order to highlight these symptoms at an early stage. This will most likely help patients to obtain an early diagnosis and line of treatment and save them from prolonged suffering.

## Abbreviations

FI: Faecal incontinence
OASI: Obstetric anal sphincter injury
UI: Urinary incontinence
